# Hand grip strength and maximum peak expiratory flow: determinants of bone mineral density of adolescent students

**DOI:** 10.1186/s12887-018-1015-0

**Published:** 2018-03-02

**Authors:** Marco Cossio-Bolaños, Cynthia Lee-Andruske, Miguel de Arruda, Cristian Luarte-Rocha, Alejandro Almonacid-Fierro, Rossana Gómez-Campos

**Affiliations:** 1 Faculty of Physical Education, State University of Campinas, Campinas, Brazil; 2 Departamento de Ciencias de la Actividad Física, Universidad Católica del Maule, Talca, Chile; 3 Universidad Nacional de San Agustín, Arequipa, Peru; 4 Red Iberoamericana de Investigación en Desarrollo Biológico Humano, Arequipa, Peru; 5 Facultad de Ciencias de la Educación, Universidad San Sebastián, Concepción, Chile; 6 Universidad Autónoma de Chile, 5 Poniente, 1670 Talca, Chile

**Keywords:** Biological maturation, Hand grip strength, Maximum expiratory flow, Bone density health, Adolescents

## Abstract

**Background:**

Maintaining and building healthy bones during the lifetime requires a complicated interaction between a number of physiological and lifestyle factors. Our goal of this study was to analyze the association between hand grip strength and the maximum peak expiratory flow with bone mineral density and content in adolescent students.

**Methods:**

The research team studied 1427 adolescent students of both sexes (750 males and 677 females) between the ages of 11.0 and 18.9 years in the Maule Region of Talca (Chile). Weight, standing height, sitting height, hand grip strength (HGS), and maximum peak expiratory flow (PEF) were measured. Furthermore, bone mineral density (BMD) and total body bone mineral content (BMC) were determined by using the Dual-Energy X-Ray Absorptiometry (DXA). Hand grip strength and PEF were categorized in tertiles (lowest, middle, and highest). Linear regression was performed in steps to analyze the relationship between the variables. Differences between categories were determined through ANOVA.

**Results:**

In males, the hand grip strength explained 18–19% of the BMD and 20–23% of the BMC. For the females, the percentage of variation occurred between 12 and 13% of the BMD and 17–18% of the BMC. The variation of PEF for the males was observed as 33% of the BMD and 36% of the BMC. For the females, both the BMD and BMC showed a variation of 19%. The HGS and PEF were divided into three categories (lowest, middle, and highest). In both cases, significant differences occurred in bone density health between the three categories.

**Conclusions:**

In conclusion, the HGS and the PEF related positively to the bone density health of both sexes of adolescent students. The adolescents with poor values for hand grip strength and expiratory flow showed reduced values of BMD and BMC for the total body. Furthermore, the PEF had a greater influence on bone density health with respect to the HGS of the adolescents of both sexes.

## Background

It is well known that peak bone mass is acquired during childhood and adolescence. This may be a key determinant of bone health and future fracture risk during adulthood [1]. A number of primary factors are believed to affect it: genetics, hormonal health, calcium intake, physical activity [2], nutritional status, vitamin E deficiency, poor calcium absorption, delayed puberty, corticosteroid use, and chronic lung diseases (cystic fibrosis) [3]. In this context, building and maintaining healthy bones during the lifetime requires a complicated interaction between a number of physiological and lifestyle factors. Therefore, the basis for bone health is created during childhood and adolescence [4].

In general, few studies have been carried out focusing on children and adolescents associating hand grip strength and maximum expiratory flow with bone density and bone mineral content [5, 6]. However, a number of studies have been conducted examining the young and adults [7–11]. These have demonstrated that hand grip strength, maximum oxygen consumption, and maximum expiratory flow are associated with bone mineral density in adults. These research studies are based on the simultaneous increase and the equivalent capacity of a muscle group to execute a simultaneous contraction on the stabilizing muscles of the trunk and extremities [5]. Therefore, during normal activity, the increased reactionary forces that control the musculoskeletal system are associated with and justified by the mechanostat theory.

During the past decade, many individuals have experienced a reduction in participating in daily physical activities (for example, walking less, greater use of cars, escalators, and elevators). Additionally, the importance of physical education and participating in sports has diminished at school and at home [12]. Consequently, these factors may play a fundamental role in the deterioration of bone health, muscle strength, and lung functioning of adolescents.

Moreover, reputable evidence exists showing that children, adolescents, and active adults have greater BMD, and they have less risk of fractures than do their inactive peers [13, 14]. Therefore, an adequate level of physical aptitude is an important contributor to bone density health, particularly, with regard to the mechanical action that promotes functional adaptation of bone [15].

Despite this, to date, few studies have been conducted examining the association between bone density health, hand grip strength, and peak expiratory flow in adolescent students. Furthermore, a systematic association could be shown between hand grip strength and maximum expiratory flow with BMD and BMC of adolescents. Thus, a reduction in respiratory function and a lower propensity for physical exercise [6] expressed in terms of strength could impair bone health in children and adolescents. Therefore, these findings examining these factors could provide relevant information about bone health for adolescents. Furthermore, adolescence is considered to be a critical stage for the acquisition of bone density [16]. During this stage, significant changes occur throughout the growth process. During biological maturation, bone density and bone mineral content must be accumulated since they can mitigate the effects and incidences of osteoporosis in the future. Therefore, to examine these factors, the objective of this study was to analyze the association of hand grip strength and maximum peak expiratory flow with the density and bone mineral content of Chilean adolescent students.

## Methods

### Sample

A descriptive correlational research study was developed. The sample studied consisted of 1427 adolescents of both sexes (750 males and 677 females) from the Maule Region from the city of Talca (Chile). Ages ranged from 11.0 to 18.9 years (15.59 ± 2.09 years). The students recruited for the study were selected from 8 schools from the public education system from the Maule Region (Chile).

Students selected for the study were those attending required physical education classes (once a week for 60 min per session) and who did not smoke. Students eliminated from the study included those engaged in training or competing in sports clubs because of their high levels of physical fitness and activity. Others excluded included those with broken bones, fractures, or a recent bone fracture.

The experimental protocol was based on the Helsinki Declaration Accord (World Medical Association for Human Subjects). In addition, the researchers obtained permission from the administration of the different schools and the ethics committee from the Universidad Autónoma de Chile. Before the study began, participants and parents were informed about the research objectives and procedures. All parents provided informed written consent authorizing their children to participate in the project.

### Procedures

To calculate age, the student’s date of birth and the evaluation date were used. Administrators from each school provided the information.

The anthropometric variables for the Dual-Energy X-Ray Absorptiometry scan, the evaluation of hand grip strength (HGS), and the Maximum Peak Expiratory Flow (PEF) were taken in a closed laboratory with temperatures between 20 to 24 °C. All measurements were taken during the months of March to July of 2015.

Standing height was measured with a portable stadimeter (Seca Gmbh & Co. KG, Hamburg, Germany) with a precision of 0.1 mm according to the Frankfurt Plan. Sitting height (celiac-trunk height) was taken using a wooden bench with a height of 50 cm with a measurement scale of 0 to 150 cm with a precision of 1 mm. All students’ anthropometric variables were measured barefoot with the least possible clothing (shirt and shorts). The body mass index (BMI) was calculated using the standard formula: body mass (kg)/height^2^ (m). Anthropometric variables were evaluated twice by three evaluators. The Technical Error of Measurement (TEM) for all of the anthropometric variables varied between 1.0 to 2.0%.

The Dual-Energy X-Ray Absorptiometry (DXA) (Lunar Prodigy; General Electric, Fairfield, CT) was used for the total body scan. Following the suggested descriptions of Kelly, Berger, and Richardson [17] and the manufacturer’s instructions, all measurements were taken by an experienced well trained technician. Before commencing the scanning process, the subject had to lie supine on their backs on the scanning platform. Arms and legs were extended parallel to the bed. Both ankles were tied together with a Velcro belt to ensure a standard position for the subjects. The BMD (g/cm^2^) and the BMC (g) of the entire body were measured in addition to the variables for the percentage of body fat (% F), muscle mass (kg), bone mass (kg), and fat mass (kg). To guarantee the reliability of the scan, the measurements were repeated right after the first ones were taken the same day for every tenth subject. The technical error of measurement (TEM) between intra-raters produced values of less than1.5%.

Biological maturation (somatic maturity status) was estimated by predicting years from attainment of age at peak height velocity. Age at peak height velocity reflects the age of maximum growth rate in stature during adolescence. Years from age at peak height velocity (APHV) were predicted for each child using sex-specific regression equations that included stature, sitting height, leg length, chronological age, and their interactions Mirwald et al. [18]. This method is simple, is non-invasive, and has demonstrated acceptable agreement when correlated against skeletal age (*r* = 0.83).

The equations used for boys included the predictive equation: Maturity Offset = − 9.236 + 0.0002708 * Leg Length and Sitting Height interaction - 0.001663 * Age and Leg Length interaction + 0.007216 *Age and Sitting Height interaction + 0.02292 * Weight by Height ratio, where *R* = 0.94, R^2^ = 0.891, and SEE = 0.592.

In girls, the predictive equation was Maturity Offset: - 9.376 + 0.0001882 *Leg Length and Sitting Height interaction + 0.0022 *Age and Leg Length interaction + 0.005841 *Age and Sitting Height interaction - 0.002658*Age and Weight interaction + 0.07693 *Weight by Height ratio, where *R* = 0.94, R^2^ = 0.890, and SEE = 0.569.

Evaluation of the Maximum Peak Expiratory Flow PEF (L/min) was conducted with the brand name device Mini Wright (Clement Clarke International Ltd., Essex, England) with a range of 60 to 900 L/min [19]. The PEF was obtained by a forced exhalation maneuver beginning with a maximum inhalation (equal to a spirometric test). Following the directions of Quanjer et al. [20], subjects were evaluated in a standing position without bending the neck. Prior to the evaluation, the device was described to the adolescent subjects, and they were allowed to practice the exercise twice (familiarization). Afterwards, the assessment was carried out with the highest value recorded of the three attempts. The Technical Error of Measurement (TEM) was less than 2% for both sexes. The PEF was classified as lowest (first tertile), middle (second tertile), and highest (third tertile).

Hand grip strength was measured with the aid of a manual hydraulic dynamometer label JAMAR (Hydraulic Hand Dynamometer® Model PC-5030 J1, Fred Sammons, Inc., Burr Ridge, IL: USA) with 0.1 lbf accuracy of both the right and left hands, following the protocol recommended by Richards et al. [21]. Each subject was seated in a straight-backed chair in the standard position. Then, each volunteer was asked to squeeze the dynamometer two times with each hand. To control for the effects of fatigue, trials on each hand alternated so that approximately 2-min rest lapsed between trials for each hand. The best value of two attempts was recorded. The inter-rater Technical Error of Measurement was less than 2.5% for both hands. HGS was classified as lowest (first tertile), middle (second tertile), and highest (third tertile).

### Statistical analysis

Data normality was verified using the Kolgomorov-Smirnov test corrected by Lilliefors, and the residue variance homogeneity was verified using the Levene test. Arithmetic descriptive statistics and the standard deviation were used to describe the variables in this study. The “t-test” of student for independent samples was used to verify the differences between the sexes. The Pearson coefficient was used to evaluate the association between the predictor variables and the dependent variables (BMD and BMC). Afterwards, linear regression analysis was performed in steps for biological maturation, hand grip strength, and maximum peak expiratory flow as the independent variables and BMD and BMC as dependent variables. Furthermore, to classify the PEF and HGS variables into categories of lowest, middle, and highest, tertiles were calculated. Differences between categories were determined through one-way ANOVA and Sheffé post hoc. In all of the cases, 0.05 was adopted as the level of significance. Statistical calculations were performed using Excel sheets and SPSS 18.0.

## Results

The descriptive statistics values for the variables for both sexes in this study are presented in Table 1 below. Females reached somatic maturation (11.78 ± 0.48 APHV) before the males (14.98 ± 0.93 APHV). Males were heavier, had a taller standing height, higher sitting height, greater % of fat, body fat, lean mass, total body BMD, total body BMC, isometric strength (right and left), and maximum peak expiratory flow in relation to the females (*p* < 0.001). No significant differences occurred in chronological age and BMI between both sexes (*p* > 0.001).

**Table 1:** Characteristics of the sample studied

Variables	Males (*n* = 750)		Females (677)		Both sexes (*n* = 1427)	
	Mean	SD	Mean	SD	Mean	SD
Age (years)	15.67	2.04	15.45	2.18	15.59	2.09
Biological age (APHV)	14.98	0.93	11.78	0.48*		
Anthropometry						
Weight (kg)	64.81	14.86	58.67	12.52*	62.66	14.38
Standing height (cm)	165.41	17.45	152.84	28.81*	160.92	22.98
Sitting height (cm)	86.93	6.17	83.02	4.28*	85.59	5.89
BMI (kg/m^2^)	23.18	4.30	23.41	4.31	23.26	4.30
Body composition (DXA)						
Percentage of Fat (% G)	23.99	8.49	34.94	7.37*	27.7	9.64
Fat Mass (kg)	15.34	8.18	20.23	8.19*	17.01	8.50
Lean Mass (kg)	46.76	9.92	36.2	6.85*	43.18	10.29
BMD (g/cm^2^)						
Body total	1.051	0.160	0.941	0.110*	1.010	0.152
BMC (g)						
Body total	2.509	0.543	2.051	0.35*	2.348	0.532
Isometric strength (lbf)						
Right Hand	65.27	30.24	50.03	19.19*	59.01	27.31
Left Hand	62.84	28.84	46.95	17.69*	56.32	26.07
Maximum peak expiratory flow (L/min)	368.89	119.37	296.35	82.86*	346.02	114.18

*X* Mean, *SD* Standard deviation, *APHV* Age at peak height velocity, *BMI* Body Mass Index, *BMD* Bone Mineral Density, *BMC* Bone Mineral Content, * = Significant difference (*p* < 0.001)

**Table 2:** Estimation of the BMD and BMC predictors base d on linear multiple regression of adolescent students

Dependent variables	Independent variables	Males				Females			
		R	R^2^	EE	p	R	R^2^	EE	p
BMD (g/cm^2^)									
Body total	Somatic Maturation	0.73	0.53	0.11	0.00	0.60	0.36	0.08	0.00
	Hand grip strength - left	0.44	0.19	0.14	0.00	0.35	0.12	0.10	0.00
	Hand grip strength - right	0.43	0.18	0.14	0.00	0.36	0.13	0.10	0.00
	Maximum peak expiratory flow	0.57	0.33	0.12	0.00	0.44	0.19	0.10	0.00
BMC (g)									
Body total	Somatic Maturation	0.79	0.62	0.31	0.00	0.64	0.41	0.24	0.00
	Hand grip strength - left	0.49	0.24	0.44	0.00	0.41	0.17	0.27	0.00
	Hand grip strength - right	0.48	0.23	0.45	0.00	0.43	0.18	0.27	0.00
	Maximum peak expiratory flow	0.60	0.36	0.41	0.00	0.44	0.19	0.30	0.00

*BMD* Mineral bone density, *BMC* Bone mineral content, *EEE* Standard Error of the Estimate

The linear regression analysis in step multiples (Tabla 2) demonstrated that the somatic maturation, left and right hand grip strength, and maximum peak expiratory flow variables were associated with the bone health (BMD and BMC) of adolescent students of both sexes. The % of explained variation was greater in males than females.

**Fig. 1:**
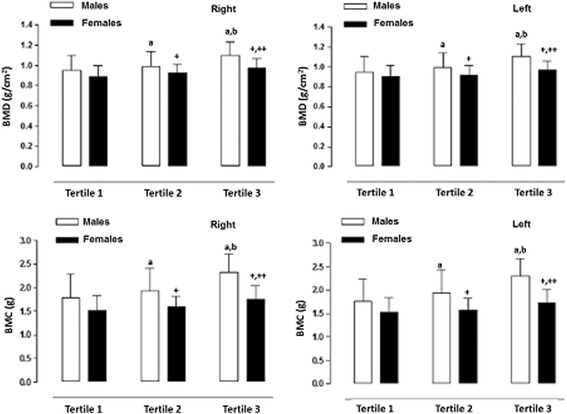
BMD and BMC values for adolescent students based on hand grip strength expressed in categories (tertiles). a: significant difference related to males in Tertile 1; b: significant difference related to males in Tertile 2; +: significant difference in females in Tertile 1; ++: significant difference in females in Tertile 2

**Fig. 2:**
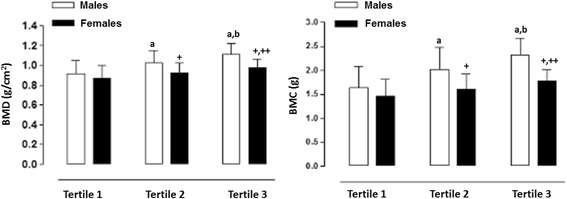
BMD and BMC values of adolescent students based on maximum peak expiratory flow expressed in categories (tertiles). a: significant difference related to males in Tertile 1; b: significant difference related to males in Tertile 2; +: significant difference irelated to females in Tertile 1; ++: significant difference related to females in Tertile 2

Figures 1 and 2 illustrate the significant differences in BMD and BMC based on the categories of PEF and hand grip strength (lowest –first tertile, middle – second tertile, and highest third tertile). Differences occurred between the three categories and in both sexes for PEF and HGS. Furthermore, males showed greater BMD and BMC when compared to the females in all of the categories.

## Discussion

The results of the research illustrate that the HGS is associated moderately to the BMD and BMC in adolescents of both sexes. These results point to a positive association between bone formation and hand grip strength in the arms. Therefore, in males, the HGS explains between 18 and 19% of the BMD and 20–23% of the BMC and in females, between 12 and 13% of the BMD and 17–18% of the BMC. These findings are consistent with those of other studies carried out on young athletes [22, 23], non-athletes [24, 25], and sedentary adults [26, 27] even though these research studies showed correlations slightly higher than those of this present study.

These results demonstrated that the PEF and the HGS independently predict bone health of this group of males and females. These systematic associations suggest that elevated levels of PEF and HGS produce a mediating effect over all the musculo-skeletal and respiratory systems. This could help promote better bone health.

The differences found in Fig. 1 are a clear illustration that the adolescents categorized here with low hand grip strength values demonstrated the lowest values in BMD and BMC in both sexes in relation to the other groups (middle and highest). In this sense, the weakness in the hand grip strength can be interpreted as bone fragility [28] in the total body. Moreover, it is associated with the loss of physical function and with a negative impact on recuperative health after an illness or surgery [29]. Furthermore, the adolescents in this study classified in the second tertile (middle) and the third tertile (highest) can be considered to have the maximum performance in hand grip strength and expiratory flow. This implies greater BMD and BMC values in both sexes.

These findings may be explained by the mechanostat theory since the bones adapt not only to static forces (of excessive weight) but also to the dynamic forces created by muscular contractions [30]. The differences in strength observed in both sexes could be explained by a greater physical performance level in males. Additionally, males tend to have greater performance levels than do females during the same stage [31]. Furthermore, females may be at greater risk of developing bone fragility during adulthood as compared to males [32].

In general, hand grip strength is associated with, at least predicts to a certain extent, bone health in adolescents in both sexes. Additionally, it might be considered as a possible indicator of bone health since it is easy to use and non-invasive for measuring muscle strength in the arms [23]. Moreover, it is suitable for clinical use in epidemiological contexts.

With regard to the maximum peak expiratory flow (PEF), the results demonstrated a positive association to bone health in both sexes. In males, the R^2^ explains 33%of the variation in the BMD and 36% in the BMC. For females, the R^2^ explains 19% in both the BMD and BMC. In fact, in some previous studies, researchers have verified that a decrease in PEF correlates to a low BMD in children and adolescents with chronic obstructive pulmonary disease (COPD) [3, 33, 34]. These results a are consistent with studies carried out in adults without COPD [10, 11].

In essence, the adolescents of both sexes in this study categorized as having lowest expiratory flow (first tertile) showed reduced values in bone health. This can be attributed to greater resistance in the respiratory airways and the dysfunction of the respiratory muscles. Therefore, the restriction of the expiratory flow can limit participation in physical exercise [6]. Consequently, it affected the expiratory flow capacity in the adolescents in this study. Therefore, individuals with low levels could be susceptible to bone disorders, including the loss of BMD. As a result, these individuals are at greater risk of bone fractures [35]. Moreover, the males performed better in terms of maximum expiratory flow compared to the females. This illustrates that males showed a greater respiratory capacity than the females. The results suggest that females should improve their PFE by engaging in physical activities and participating in basic programs to improve muscle strength in physical education classes. In the long run, these appear to be valid strategies for reducing the number of falls and fractures [36].

The results from this research suggest that the greater the flow of air exhaled, the greater the lung capacity to carry out physical exercise. Therefore, childhood and adolescence are critical stages for solidifying the practice of physical activity. Studies suggest developing cardio-pulmonary capacity [37, 38] and physical activity in general for improving bone health in children and adolescents. Furthermore, other studies have demonstrated that the expiratory flow is associated with good physical functioning, cognition, and mortality in adult subjects of an advanced age [39, 40]. However, it is important to reiterate that the association between lung functioning and BMD are complicated, and they are still not clear [41].

In essence, the practice of moderate physical activity, especially, strenuous activities that involve the support of body weight and the playing of sports, are important for promoting bone strength [42, 43]. Moreover, researchers suggest maximizing lung function to optimize bone health in adolescents.

In general, the PEF is one of the important parameters developed for evaluating lung function and for diagnosing, managing, and following respiratory diseases [44]. Moreover, its use and application is justified not only in children and adolescents with asthma but also with student populations without apparent pulmonary disorders.

It is important to point out that the present research has a few limitations. In the first place, the type of transversal study did not allow a causal association to be established. However, future studies need to include experimental and/or longitudinal research to establish causality between the variables examined. In the second place, the associations analyzed here are probably influenced by other intervening variables such as the quantity consumed of vitamin D and the cardio-pulmonary aptitude level. These variables were not available; however, if they would have been, they would have allowed us to discuss the results more effectively. On the other hand, with regard to the strength of the study, it is important to reiterate that the sample size was ample and representative of adolescent students participating. Moreover, the assessment of bone health by using the “Gold Standard”: the DXA and the easy to access and easy to use devices (the manual dynamometer and the Mini Wright device). These are strong points of this study as is the control for biological maturation. These points make this study applicable to other student contexts.

## Conclusion

In conclusion, the HGS and the PEF are associated positively to bone health of adolescent students of both sexes. The students with lowest hand grip strength and expiratory flow values showed lower values of BMD and BMC. Moreover, it is important to point out that the influence of the PEF is greater with regard to bone health with respect to muscle strength in the arms of adolescents. These results reinforce inclusion of physical exercise to improve lung functioning and muscle strength in adolescent students. However, more studies are needed focusing on adolescent students to confirm our findings.

## Abbreviations

APHV: Age at peak height velocity
BMC: Body bone mineral content
BMD: Bone mineral density
BMI: Body Mass Index
DXA: Dual-Energy X-Ray Absorptiometry
HGS: Hand grip strength
PEF: Maximum peak expiratory flow
TEM: Technical error of measurement
